# Cases Admitted into the Surgical Wards of the Hospital Since October 20, 1844, (73 Cases)

**Published:** 1845-01

**Authors:** F. W. Sargent

**Affiliations:** Resident Surgeon


### PENNSYLVANIA HOSPITAL.

### *Surgical Wards.*

Service of Dr. Randolph.

*Cases admitted into the Surgical Wards of the Pennsylvania Hospital, since October* 20, 1844.

Abscesses in various parts, 4; burn, 2, (died 1); calculus in bladder, 3; calculus in urethra, 1; contusion of deltoid, 1; contusion of other parts, 8; coxalgia, 2; dislocation of ulna backwards, 1; dislocation of elbow backwards, 1; fracture, compound, of skull, 1; fracture, compound, of humerus, 1; fracture, compound, of hand, 1; fracture, compound, of finger, 3; fracture, compound, of thigh, &c., 1, (died 1); fracture, compound, of leg, 2, (died 1); fracture, simple, of spine, 1; fracture, simple, of jaw, 2; fracture, simple, of clavicle, 3; fracture, simple, of forearm, 1; fracture, simple, of radius, 2; fracture, simple, of thigh, 2; fracture, simple, of ribs, 1; hydrocele, 1; inflammation of knee, 2; inflammation of foot, 3; stricture of urethra, 1; syphilis, 7; tumour, œtheromatous, 1; tumour, fatty, 1; ulcer, 5; wound, gunshot, of neck, 1, (died, 1); wound, gunshot, of thigh, 1; wound, gunshot, of scalp, 1; wound, gunshot, of hand, 1; wound, punctured, of chest, 2; wound, lacerated, of chest, 2.—Total admissions, 73; deaths, 4.

Number of operations, 7.

Lithotomy, 2, (died, 1*); amputation of forearm, 1; amputation of arm, 1; removal of tumours, 2; ligature of common carotid, 1, (died, 1.)

The case of dislocation of the ulna was complicated with fracture of the coronoid process. The arm was in a position be-

* Died of peritonitis, 48 hours after the operation. The patient was a child, aged four years.

tween that of complete extension and flexion. The dislocation was easily reduced by drawing upon the forearm in the direction assumed by the limb, (the humerus being fixed,) and then gradually flexing it.

In the other case of dislocation, the elbow was thrown backwards. The diagnosis was perfectly easy, as was also the reduction of the injury, which was accomplished in the same manner as the first. In this case there was no fracture. The patient, while very much intoxicated, was thrown in wrestling, striking the ground upon his hand, the forearm being flexed. Both patients are still under treatment, and are likely to recover with very little or no imperfection in the motions of the elbow.

The following case of gunshot wound was a very interesting one to us.

A man, aged 22, in perfect health, was accidentally shot, Nov. 1, with a pistol which was loaded only with powder and wadding. The load took effect upon the cheek and upper part of the neck of the right side. The wound extended in one direction about three inches, and in the other, two inches. The lower jaw was fractured at its angle, and the bone stripped of its periosteum from a little above the angle to the symphysis. The soft parts were very much mangled, and a communication made between the cavity of the mouth, exteriorly to the alveolar process of the jaw, and the wound in the neck. There was little or no bleeding at the time; the temporal artery was felt pulsating, and the carotid about ½ inch on the outer side of the wound, just below the angle of the jaw. Several small fragments of bone and remnants of the wadding were removed from the wound, which was covered with a warm poultice, and supported by a bandage, and the patient was kept under the influence of opium.

Nov. 7. Symptoms have all been favourable—no fever, no pain, excepting a slight soreness in swallowing. The wound becoming clean. Hypoglossal nerve seen at the bottom of the wound, having been divided at a point a little anterior to its curve. Considerable induration of the surrounding parts.

Same treatment continued as above. Diet farinaceous.

8. Commenced bleeding at 8 o'clock, A. M., and bled profusely. Hæmorrhage not controlled by pressure at the root of the neck, and it was prevented from appearing at the wound by pressure made at this point, it came from the mouth. In this emergency, Dr. Peace tied the carotid low down in the neck, and then found it necessary to apply a ligature upon a small artery, probably the facial, in the wound. The external jugular vein was cut during the operation, and a fine ligature was applied around the orifice, the vein not having been cut completely across.

The operation was not followed by any unpleasant symptom whatever.

12th. Lost a few ounces of blood this morning from some small vessel; the bleeding stopped, however, after the wound had been for a few minutes exposed to the air.

14th. A small artery, probably a branch of the facial or labial of the left side, poured out ℥ij. a ℥iii. of blood; and the coats of the vessel were so much softened, by inflammation, that we were unable to apply a ligature: pressure was substituted.

15th. The ligatures came away from the vein and from the small artery which was tied at the same time.

19th. Ligature loosened from the carotid and came away with the dressings. The wound made in tying the vessel did not close by the first intention—its granulations firm. The original wound contracting very finely, diminished to one-third its former size—only half inch of the bone exposed. Communication between the mouth and the wound closed. General symptoms most favourable. The most perfect rest has been enforced; the patient has been constantly watched day and night; mild and nutritious diet; opiates occasionally.

28th. A few ounces of blood lost from the wound made in tying the carotid. It was of a bright red colour, and came in jets from the little gutter, as it were, made by the ligature—it was easily arrested by very gentle pressure made just below the inferior extremity of the wound, and upon the vessel itself; the precise source of the bleeding could not be detected, and it appeared doubtful whether the jet was owing to the direct passage of the blood from an artery, or merely to the succussion caused by the large vessels.

Small losses of blood occurred again on the 30th, and on the 2d, 4th and 5th of December: the bleeding was very readily arrested in the same manner as before, viz., by pressure made at the lower extremity of the wound. The granulations had now become flabby, and the wound indisposed to heal, but the reparative process was still going on at the original wound.

6th. Very profuse bleeding: the stream so strong and large that we had now no doubt that the carotid was opened both above and below the point of ligature. Very firm pressure now was requisite to control the flow of blood. The wound had become much enlarged from sloughing; the parts beneath were softened and easily broken down, and emitted a gangrenous smell. Hæmorrhage occurring in the evening, some powdered matico was thrust into the wound and pressure made upon it. Slight hæmorrhage on the 8th and on the morning of the 9th. Died in the evening of the 9th.

*Post Mortem Appearance.*—The original wound contracted to about the size of a twenty-five cent piece. No union of the fractured extremities of the bone. That part of the bone which was still exposed was not at all loosened from the body of the jaw. The wound made in tying the vessel, three inches long and two inches wide. Surrounding integuments thin, and of a purple colour. Wound filled with a soft, black and fœtid coagulum. The integuments were turned off from the right side of the neck, in order to give a fair view of the part beneath. The sterno-cleido mastoid muscle in the lower half of its extent was partially removed by sloughing. The clot possessing the characters above mentioned was reposing on the transverse processes of the vertebræ, and bounded internally by the trachea, externally by the remains of the mastoid muscles. The clot was carefully removed from the parts by pouring water upon them, and in this manner we were able to obtain a view of the vessels. The common carotid, at a point an inch and a half above its origin, was open, and at this point we supposed that the ligature was applied—it seemed as if the vessel had been cut completely through; the extremity was slightly irregular on its edges, and all the coats thickened—while its calibre was much diminished by the deposit of lymph on its internal membrane—still it readily permitted the passage through it of a silver director of the ordinary size—the point at which the calibre of the vessel had been most diminished was about half an inch from the free extremity. The superior portion of the vessel was discovered by cutting down upon it at the upper part of the neck and passing a probe through it. Between the two extremities of the vessel was a space of three quarters of an inch, in which the vessel had been destroyed and removed by sloughing. The lower extremity of the inferior portion of the vessel was soft, black and gangrenous, and this condition extended to within an inch of its bifurcation. This portion of the vessel contained a soft and recent coagulum; the lower portion of the artery contained no clot. Both extremities, therefore, of the common carotid must have allowed free exit to the blood. The coats of the internal jugular vein were entire, but were stained of a dark brown colour.

F. W. Sargent, M. D.,
*Resident Surgeon.*

*December* 21, 1844.

[*Medical News.*

